# Postoperative acute kidney injury can be predicted by the novel biomarkers insulin-like growth factor-binding protein 7/tissue inhibitor of metalloproteinases-2 as early as 6 hours after surgery

**DOI:** 10.1186/cc13569

**Published:** 2014-03-17

**Authors:** I Göcze, R Herzog, M Koch, P Renner, F Zeman, BM Graf, HJ Schlitt, T Bein

**Affiliations:** 1 University Medical Center Regensburg, Germany

## Introduction

Acute kidney injury (AKI) in surgical critically ill patients is an independent risk factor for early mortality. Two novel urine biomarkers, insulin-like growth factor-binding protein 7 (IGFBP7) and tissue inhibitor of metalloproteinases-2 (TIMP-2), may help to detect clinically silent episodes of AKI in the golden hours prior to irreversible damage of the kidney. We evaluated the early predictive value of these biomarkers for AKI, moderate and severe AKI, early requirement of renal replacement therapy (RRT), and ICU mortality, with a cutoff value of IGFBP7/TIMP-2 >0.3.

## Methods

Four to six hours after admission to the surgical ICU, urine biomarkers were prospectively evaluated in all patients with present exposures and susceptibilities for AKI according to the KDIGO recommendation. The incidence and severity of AKI (KDIGO 2012) and requirement of RRT were assessed over 48 hours after admission. In addition, ICU mortality and variables such a norepinephrine dose, mean arterial pressure, hemoglobin level, cumulative fluid balance and urine production were noted at the time of biomarker evaluation (4 to 6 hours) and for the first 24 hours after admission.

## Results

A total of 120 patients were included in the study. The area under the curve (AUC) for IGFBP7/TIMP2 >0.3 was 0.83 for early detection of AKI, 0.86 for moderate and severe AKI, 0.86 for RRT in the first 48 hours after admission and 0.86 for ICU mortality. Patients with IGFBP7/ TIMP-2 >0.3 had a higher risk (odds ratio 11.7) for development of AKI compared with patients with IGFBP7/TIMP-2 ≤0.3. The norepinephrine dose, mean arterial pressure, fluid balance and urine output in the first 6 hours after admission were significantly different for patients with low and high risk for AKI. The negative predictive value (NPV) of 0.41 for urine output <1 ml/kg/hour over the first 6 hours after admission was significantly lower if compared with NPV of 0.86 for GFBP7/TIMP-2 ≤0.3 for exclusion of AKI. Similarly NPV of urine output <1 ml/kg/hour of 0.89 for moderate and severe AKI (stage 2 and 3) was lower than the NPV of 1.00 for GFBP7/TIMP-2 ≤0.3.

## Conclusion

The novel urine biomarkers IGFBP7 and TIMP-2 enable early risk stratification of surgical ICU patients at risk for development of AKI. The predictive values of biomarkers were better for early prediction than clinical parameters such as urine output within the first 6 hours after admission to ICU.

